# The blood–brain barrier significantly limits eflornithine entry into *Trypanosoma brucei brucei* infected mouse brain^1^

**DOI:** 10.1111/j.1471-4159.2008.05706.x

**Published:** 2008-11

**Authors:** Lisa Sanderson, Murat Dogruel, Jean Rodgers, Barbara Bradley, Sarah Ann Thomas

**Affiliations:** * King’s College London, Pharmaceutical Sciences Research Division, Hodgkin BuildingLondon, UK; † Department of Veterinary Clinical Studies, University of Glasgow Veterinary SchoolGlasgow, UK

**Keywords:** Blood–brain barrier, choroid plexus, eflornithine, trypanosomiasis

## Abstract

Drugs to treat African trypanosomiasis are toxic, expensive and subject to parasite resistance. New drugs are urgently being sought. Although the existing drug, eflornithine, is assumed to reach the brain in high concentrations, little is known about how it crosses the healthy and infected blood–brain barrier. This information is essential for the design of drug combinations and new drugs. This study used novel combinations of animal models to address these omissions. Eflornithine crossed the healthy blood–CNS interfaces poorly, but this could be improved by co-administering suramin, but not nifurtimox, pentamidine or melarsoprol. Work using a murine model of sleeping sickness demonstrated that *Trypanosoma brucei brucei* crossed the blood–CNS interfaces, which remained functional, early in the course of infection. Concentrations of brain parasites increased during the infection and this resulted in detectable blood–brain barrier, but not choroid plexus, dysfunction at day 28 post-infection with resultant increases in eflornithine brain delivery. Barrier integrity was never restored and the animals died at day 37.9 ± 1.2. This study indicates why an intensive treatment regimen of eflornithine is required (poor blood–brain barrier penetration) and suggests a possible remedy (combining eflornithine with suramin). The blood–brain barrier retains functionality until a late, possibly terminal stage, of trypanosoma infection.

Human African trypanosomiasis (HAT) or sleeping sickness is caused by the parasites *Trypanosoma brucei* (*T.b.*) *gambiense* or *T.b. rhodesiense* and is fatal if untreated. The first stage following infection corresponds to trypanosome proliferation in the peripheral system. The disease enters the second stage when the parasites establish within the CNS. Once within the CNS, the parasites are considered protected against pentamidine and suramin (stage 1 drugs) by the blood–CNS interfaces (Raseroka and Ormerod 1986; Bronner *et al.* 1991; Balasegaram *et al.* 2006; Sanderson *et al.* 2007). These consist of the blood–brain barrier (BBB), located at the cerebral capillary endothelium, and the blood–CSF barrier, formed by the choroid plexuses and arachnoid membrane. Hence HAT treatment is stage-specific and it is assumed that the stage 2 drugs, melarsoprol and eflornithine, must cross the blood–CNS barriers well (Masocha *et al.* 2007; Priotto *et al.* 2008). Melarsoprol is a toxic arsenical derivative which causes a post-treatment reactive encephalopathy in 5–10% of patients, resulting in a case-fatality rate of ∼50% (Kennedy 2004). Furthermore, an increase in melarsoprol treatment failure has been noted (Brun *et al.* 2001). Eflornithine, an ornithine decarboxylase inhibitor, is active against second stage *T.b. gambiense*, but not *T.b. rhodesiense,* because of its high ornithine decarboxylase turnover (Iten *et al.* 1997). Studies indicate that eflornithine is safer than melarsoprol against *T.b. gambiense* HAT (Chappuis *et al.* 2005; Balasegaram *et al.* 2006; Priotto *et al.* 2008). Nevertheless, eflornithine monotherapy is not a long-term solution. This regimen is expensive, complicated to administer, less effective in HIV-endemic areas and could cause parasite resistance. New drug candidates, especially for stage 2, are urgently required. In addition to CNS efficacy models, emphasis has been placed on screening compounds for their ability to cross the BBB. However, it is notoriously difficult to deliver drugs to the CNS. In fact, discovery programmes aimed at identifying drugs for CNS disorders are more likely to fail than programmes in other therapeutic areas (Reichel 2006). New molecules against late-stage HAT are unlikely to be available in the near future. Alternative approaches have been to optimize the existing drug regimens and to develop combination chemotherapies to improve efficacy. Trials have been conducted using eflornithine with melarsoprol or nifurtimox, which is available for compassionate use against HAT. Although, the eflornithine-melarsoprol trial was terminated because of excessive fatalities, the eflornithine–nifurtimox combination remains promising (Priotto *et al.* 2007). This improved efficacy may be due to increased CNS drug entry, but this has not been investigated. This lack of knowledge regarding the underlying synergistic mechanisms means that the full potential of drug combinations is not being exploited (Burri and Brun 2003).

Despite its importance in understanding anti-trypanosomal drug delivery, the function of the blood–brain and blood–CSF barriers throughout the course of trypanosomiasis is unknown. Early localization of the parasites to the circumventricular organs (CVOs), meninges and CSF has been reported (Jennings and Gray 1983; Schmidt 1983; Schultzberg *et al.* 1988; Keita *et al.* 1997). BBB damage occurs in the late-stage of *T.b. brucei* rodent infections (Philip *et al.* 1994) and transient increases in paracellular permeability co-ordinate with *T.b. rhodesiense* movement across the human BBB *in vitro* (Grab *et al.* 2004; Nikolskaia *et al.* 2008). An understanding of eflornithine distribution in the healthy and infected CNS is essential if we are to improve efficacy, reduce toxicity and design new drug combinations. Investigating the effects of infection on barrier integrity should also clarify the importance of CNS delivery for HAT drugs.

In this study, we used well-established methods in combination: the sensitive *in situ* brain/choroid plexus perfusion method and a murine model of late-stage HAT (Jennings *et al.* 2002; Sanderson *et al.* 2007). Studies explored the pharmacokinetic characteristics of eflornithine transport across the healthy blood–CNS interfaces both alone and with other anti-trypanosomal agents. The potential removal of eflornithine by the BBB efflux transporter, P-glycoprotein (P-gp), was also investigated using P-gp-deficient and wild-type mice. Further studies explored eflornithine drug delivery and blood–CNS barrier integrity at set time-points in mice that had been infected with *T.b. brucei* GVR 35 and attempted to correlate this with parasite existence within the CNS.

## Materials and methods

### Materials

[^3^H]Eflornithine hydrochloride was custom radiolabelled (500 mCi/mmol, radiochemical purity 97.6%; Moravek, CA, USA). [^14^C]Sucrose (498 mCi/mmol, radiochemical purity ≥ 98%) and d-[1-^3^H(N)]-mannitol (14.2 Ci/mmol, radiochemical purity > 97%) were purchased [Moravek and Perkin-Elmer (Boston, MA, USA), respectively]. Eflornithine hydrochloride, suramin, and pentamidine isethionate sodium salt were purchased from Sigma (Dorset, UK). Nifurtimox and melarsoprol were a gift (Professor Croft; London School of Hygiene and Tropical Medicine, UK).

### Animals

Procedures were performed within the animal scientific procedures act (1986) and specified animal pathogen order (1998) guidelines. BALB/c mice were purchased (Harlan; Oxon, UK). A breeding colony of FVB-Mdr1a/1b(+/+) and FVB-Mdr1a/1b(−/−) mice (Taconic; NY, USA) was established and genotype confirmed by PCR analysis (Harlan). Dr. Alfred Schinkel (Netherlands Cancer Institute) is the creator of Mdr1a/1b mice. Mice were maintained under standard temperature/lighting conditions and given food and water *ad libitum*.

### Characterization of *T.b. brucei* GVR 35 infection in BALB/c mice

A murine model of HAT which uses cloned stabilates of *T.b. brucei* GVR 35 to infect outbred CD1 mice is established and exhibits many of the human neuropathological changes (Jennings *et al.* 2002). In the present study, *T.b. brucei* GVR35/C1.7 was passaged through inbred BALB/c mice. Parasitaemia was estimated using the ‘Matching Method’ (Herbert and Lumsden 1976). At peak parasitaemia, blood was obtained by cardiac puncture, preserved under liquid nitrogen and was used to infect all the mice. Mice were infected by i.p. injection of 2 × 10^4^ trypanosomes diluted in 0.1 mL phosphate-buffered saline containing 15 g/L glucose pH8.0 (PBS-G) and the mean survival time calculated. Parasitaemia was monitored, using an improved Neubauer haemocytometer, in venous blood collected using heparin-flushed (1000 U/mL) red blood cell pipettes and diluted 1 : 100 with PBS-G. In CD1 mice, the CNS-stage of the disease is reached between 14 and 21 days post-infection (p.i.) (Jennings and Gray 1983). To determine when the parasites establish within the CNS in the BALB/c strain, groups of 3–7 mice were infected and parasitaemia monitored. The mice were treated with diminazene aceturate [Berenil® Hoechst (Frankfurt, Germany), 40 mg/kg i.p.], on day 7, 11, 12, 13, 14 or 21 p.i. (Jennings and Gray 1983). Seven days after Berenil treatment, blood from each mouse was examined to confirm the absence of systemic trypanosomes. Mice were anaesthetized (i.p. medetomidine hydrochloride (2 mg/kg) and ketamine (150 mg/kg)) and perfused via the heart with sterile saline. Each mouse brain was excised, homogenized in 1 mL sterile saline and injected i.p. into a naïve recipient mouse. Whole blood from the tail vein of recipient mice was checked daily for parasitaemia up to 28 days p.i. (Jennings *et al.* 1979, 2002). Since Berenil is a first stage trypanocidal drug, this treatment will not cure infections that have progressed to the CNS. Therefore, any trypanosomes detected in the recipient mice have been transferred in the brain homogenate indicating the presence of CNS disease in the original animals (Jennings *et al.* 1979).

### *In situ* perfusion technique

The heart perfusion method was used as described by Sanderson *et al.* (2007). Adult male mice (∼25 g) were anaesthetized as above and heparinized (100 U, i.p.). Flow rate was 5 mL/min for up to 60 min. After perfusion, a cisterna magna CSF sample was taken, the animal decapitated and brain removed. Samples of frontal cortex, occipital cortex, caudate putamen, hippocampus, hypothalamus, thalamus, pons, cerebellum, fourth ventricle choroid plexus, pineal and pituitary glands were taken. These regions were selected as they are affected by trypanosomes (Sanderson *et al.* 2007). All the brain matter remaining after these samples had been taken, underwent capillary depletion analysis (Sanderson *et al.* 2007). In brief, a brain homogenate was prepared using a buffer and a dextran solution. Final dextran concentration was 13%. Centrifugation of this homogenate produced an endothelial cell-enriched pellet and a brain parenchyma containing supernatant. Capillary depletion (including brain homogenate, supernatant and pellet), brain regions, CVOs, CSF and plasma samples were solubilized by 3.5 mLs Solvable (Perkin-Elmer; 0.5 mLs). Lumasafe scintillation fluid (Perkin-Elmer) was then added. Sample radioactivity was quantified (Packard Tri-Carb 2900TR counter).

### Experimental design

#### Multiple-time experiments

Heart perfusions were performed in BALB/c mice. [^3^H]eflornithine (1 μM) and [^14^C]sucrose (1 μM) were present in the artificial plasma for up to 30 min. [^14^C]sucrose is a baseline marker. In brain samples, it measured vascular space. Any deviation from the norm indicated loss of BBB integrity or under-perfusion of the tissue. In pineal and pituitary gland samples, [^14^C]sucrose measures vascular space and reflects the ability of [^14^C]sucrose to cross between endothelial cells. Thus, also provides a measure of the paracellular permeability characteristics of these tissues. In choroid plexus, [^14^C]sucrose, additionally represents the extracellular space formed between the choroidal capillary endothelium and epithelium.

#### Effect of drug on barrier integrity

The effect of prolonged eflornithine (250 μM) exposure on barrier integrity was observed by perfusing BALB/c mice for 60 min. In the final 10 min [^3^H]mannitol (0.035 μM) and [^14^C]sucrose (1 μM) were also included in the artificial plasma. [^3^H]mannitol also measures vascular and extracellular space. However, its small size (MW182) allows it to be more sensitive than [^14^C]sucrose (MW342) to alterations in barrier integrity. Results were compared with mice that were perfused in the absence of eflornithine.

#### Transporter investigations

To investigate the role of P-gp on eflornithine transport, FVB-Mdr1a/Mdr1b(+/+) and FVB-Mdr1a/Mdr1b(−/−) mice were perfused (30 min) with [^3^H]eflornithine (1 μM) and [^14^C]sucrose (1 μM). To determine whether eflornithine distribution was affected by a saturable system, 10 min perfusions were performed using BALB/c mice and artificial plasma containing [^3^H]eflornithine (1 μM) and [^14^C]sucrose (1 μM) and unlabelled anti-trypanosomal drug at concentrations comparable to those measured in the plasma of treated patients [either 250 μM eflornithine, 150 μM suramin, 200 μM suramin (Milord *et al.* 1993), 10 μM pentamidine (Waalkes and DeVita 1970), 30 μM melarsoprol or 6 μM nifurtimox (Gonzalez-Martin *et al.* 1992)]. Each experiment also involved a pre-isotope perfusion period of 10 min. During this pre-isotope perfusion, the plasma also contained the unlabelled drugs. Control experiments mirrored these experiments except no unlabelled drugs were present throughout the perfusion.

#### Effect of parasite on barrier integrity/permeability

To investigate the effects of infection on barrier integrity and drug penetration BALB/c mice were infected with *T.b. brucei* as described. At day 7, 14, 21, 28 and 35 p.i., groups were *in situ* perfused with [^3^H]eflornithine (1 μM) and [^14^C]sucrose (1 μM) for 30 min. The fourth ventricle choroid plexus weight was monitored.

### Expression of results

Tissue radioactivity (dpm/g) was expressed as a percentage of that in plasma (dpm/mL) and termed *R*_Tissue_ (mL/100 g). Where stated, the *R*_Tissue_ for eflornithine has been corrected for vascular/extracellular space by subtraction of the [^14^C]sucrose *R*_Tissue_ value. Blood-to-brain unidirectional rate constants (*K*_in_) were determined by single-time uptake analysis (*K*_in_ = [^14^C]sucrose corrected *R*_Tissue_ values/perfusion time) after 30 min perfusions (Williams *et al.* 1996).

### HPLC analysis

To ensure the integrity of [^3^H]eflornithine during passage through the cerebral circulation, samples of arterial inflow and venous outflow, collected at 10, 20 and 30 min, were analysed by HPLC (Jasco, Essex, UK). Samples (100 μL) were passed through a Hamilton PRP-X300, 7 μm (250 × 4.1 mm) column using 0.3 mL/min 30 mM potassium dihydrogen phosphate buffer (adjusted to pH2.2 with 85% orthophosphoric acid) and acetonitrile (50 : 50) over 35 min (Hanpitakpong *et al.* 2003). The column eluant was then mixed 1 : 3 with scintillation fluid (UltimaFlo M; Perkin-Elmer) in a radioactive detector (Packard, UK) to allow real-time radioactive analysis.

### Octanol-saline partition coefficient and protein binding

An octanol-saline partition coefficient (pH 7.4) was determined for [^3^H]eflornithine (0.67 μM). Binding of [^3^H]eflornithine (0.4 μM) to murine (male FVB) and human (Sigma) plasma proteins and to the artificial plasma bovine serum albumin and dextran, was measured using Centrifree micropartition devices (MA, USA) (Sanderson *et al.* 2007). Lyophilized human plasma was reconstituted in 1 mL deionized water.

### Data Analysis

Comparisons were made between appropriate groups and differences at the 5% level considered significant. Two-way anova followed by Tukey’s multiple range test was employed in each case using Sigma Stat software (SPSS Software Ltd, Birmingham, UK). Means, SEM and *p*-values are provided as summary statistics.

## Results

### Multiple-time studies

**Fig. 1:**
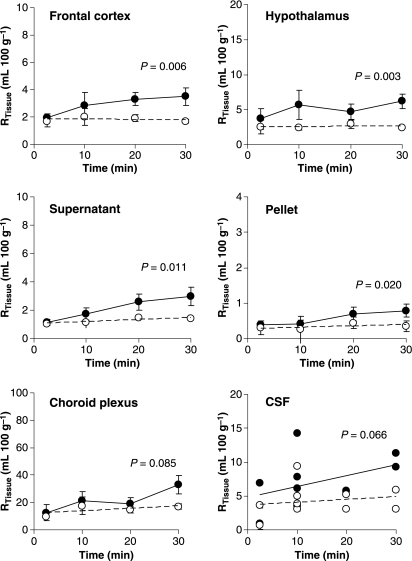
*R*_Tissue_ values for [^3^H]eflornithine (---•---) and [^14^C]sucrose (--○--) in selected brain regions, capillary depletion, choroid plexus and CSF samples plotted against perfusion time. Supernatant and pellet were obtained from capillary depletion analysis of brain homogenate. Values are mean ±SEM (*n* = 3–6), except for CSF where individual points are plotted because of the limited number of samples.

No differences in [^14^C]sucrose/vascular space values (*R*_Tissue_) were observed between the frontal cortex, caudate putamen, occipital cortex, hippocampus, hypothalamus and thalamus (Fig. 1). Values in these regions ranged from 1–2 mL/100 g (30 min). This was smaller than that measured in pons and cerebellum (∼3 mL/100 g; *p* < 0.001). These results are comparable to published values (Sanderson *et al.* 2007). [^3^H]eflornithine *R*_Tissue_ values were higher than those for [^14^C]sucrose (Fig. 1; *p* < 0.01 for all brain regions except the caudate putamen, where the difference did not attain statistical significance). When corrected for vascular space, the *R*_Tissue_ values for [^3^H]eflornithine reached only 1–4 mL/100 g in the brain at 30 min (equivalent to 10–40 nM or 0.003–0.011% of administered dose). The [^14^C]sucrose-corrected distribution of [^3^H]eflornithine over time was higher in the hypothalamus (*K*_in_; 1.3 ± 0.4 μL/min/g) when compared with the caudate putamen (0.5 ± 0.2 μL/min/g) and hippocampus (0.5 ± 0.2 μL/min/g) (*p* = 0.021 and *p* = 0.043, respectively), but not to other regions including frontal cortex (0.6 ± 0.2 μL/min/g), occipital cortex (0.6 ± 0.2 μL/min/g), pons (1.2 ± 0.4 μL/min/g), cerebellum (1.0 ± 0.3 μL/min/g) and thalamus (0.9 ± 0.2 μL/min/g).

[^3^H]eflornithine was detected in brain homogenate, supernatant and capillary endothelial cell-enriched pellet at higher *R*_Tissue_ levels than [^14^C]sucrose (Fig. 1; *p* = 0.001, *p* = 0.011 and *p* = 0.02 respectively). When corrected for [^14^C]sucrose space, the distribution of [^3^H]eflornithine was only 2.0 ± 0.8, 1.6 ± 0.5 and 0.4 ± 0.2 mL/100 g respectively after 30 min (equivalent to 20, 16 and 4 nM or 0.005, 0.004 and 0.001% of administered dose, respectively).

[^3^H]eflornithine *R*_Tissue_ in the pineal and pituitary glands was higher than that achieved for [^14^C]sucrose (*p* = 0.002 and 0.023 respectively). When corrected for vascular space, [^3^H]eflornithine distribution reached 48 ± 16 and 26 ±11 mL/100 g in the pineal and pituitary glands respectively (30 min). Overall, [^3^H]eflornithine levels measured over time in the choroid plexus were not different to those achieved for [^14^C]sucrose, even though at 30 min [^3^H]eflornithine reached 16 ± 6 mL/100 g ([^14^C]sucrose corrected; *p* = 0.085; Fig. 1). [^3^H]eflornithine and [^14^C]sucrose distribution into CSF was measured after a 10 min perfusion at 7.9 ± 2.3 and 5.3 ± 1.4 mL/100 g, respectively (*R*_Tissue_, *n* = 4). There was no difference between the CSF concentration of [^3^H]eflornithine compared to [^14^C]sucrose over time (Fig. 1). No differences were observed between the HPLC chromatograms obtained from the arterial and venous samples collected – data not shown.

### Effect of eflornithine on barrier

No differences were observed for the vascular markers, [^14^C]sucrose and [^3^H]mannitol, in any brain, capillary depletion or CVO samples when 250 μM unlabelled eflornithine was present and compared with controls (*p* > 0.05 for each marker and sample type)-data not shown.

### Transporter investigations

No differences were observed in the *R*_Tissue_ values achieved for [^3^H]eflornithine or [^14^C]sucrose for the brain, capillary depletion samples and CVOs from FVB-mdr1a/1b(−/−) compared with FVB-mdr1a/1b(+/+) mice-data not shown. Since [^3^H]eflornithine could cross the BBB and be detected in the brain, experiments were performed with unlabelled eflornithine to investigate whether [^3^H]eflornithine was subjected to uptake or efflux by a saturable mechanism. Control studies revealed that the pre-isotope perfusion had no effect on the percentage of either [^14^C]sucrose or [^3^H]eflornithine detected in brain regions. In addition, no differences were observed in the vascular space or the *R*_Tissue_ values of [^3^H]eflornithine detected in the absence or presence of 250 μM unlabelled eflornithine in the artificial plasma – (*n* = 3–7 for each group; data not shown).

### Effect of drug combinations on [^3^H]eflornithine distribution

Both 150 and 200 μM suramin had no effect on the [^14^C]sucrose *R*_Tissue_ values in any sample. The sucrose-corrected distribution of [^3^H]eflornithine into the brain and CVOs was increased with both suramin concentrations when compared with controls (Table 1: *p* < 0.001 for each concentration). There was a difference in the sucrose-corrected [^3^H]eflornithine distribution into the brain (*p* < 0.001), but not the CVOs, between the 150 and 200 μM suramin groups. An increase in [^3^H]eflornithine distribution ([^14^C]sucrose corrected) into all capillary depletion samples in the presence of suramin was also observed (*p* = 0.006 for each concentration). This increase was most evident in the endothelial cell-enriched pellet (Table 1). No difference was observed between the two concentrations for the capillary depletion samples.

**Table 1:** Percentage increase in [^3^H]eflornithine distribution ([^14^C]sucrose-corrected) observed in the presence of suramin

	% Increase in *R*_Tissue_ values of [^3^H]eflornithine	
Region	+150 μM suramin	+200 μM suramin
Frontal cortex	36.4 ± 9.9	46.9 ± 11.0
Hypothalamus	51.2 ± 15.2	170.6 ± 26.0
Pons	59.5 ± 13.6	74.4 ± 17.2
Homogenate	42.2 ± 9.1	58.0 ± 15.2
Supernatant	14.2 ± 13.6	11.8 ± 11.9
Pellet	838.9 ± 386.8	842.6 ± 242.1
Choroid plexus	639.5 ± 85.9	1085.2 ± 497.3

Values obtained in the presence of suramin were all significantly higher compared to control. Each group *n* = 4–8.

No differences were observed in the concentrations of either [^14^C]sucrose or [^3^H]eflornithine in any brain regions, capillary depletion samples or CVOs sampled when either unlabelled pentamidine, nifurtimox or melarsoprol were added to the artificial plasma (*p* > 0.05 for each isotope and drug) – data not shown.

### Entry of parasites into the brain

**Fig. 2:**
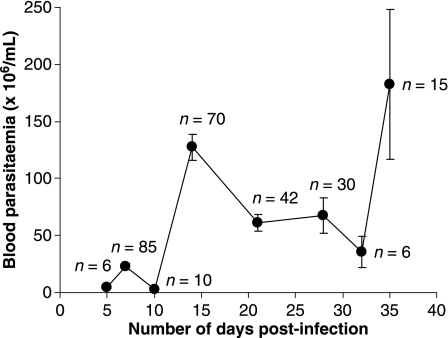
Whole blood parasitaemia in BALB/c mice after infection with stabilate GVR 35. Mean ± SEM (*n* = 6–85). Sample size varied because of amalgamation of all laboratory results to achieve the most accurate picture of the fluctuations. Average survival time was 37.9 ± 1.18 days.

Parasites were detected in the blood from day 3 and this was quantifiable using an improved Neubauer haemocytometer from day 4–5 (Fig. 2). Mice were observed daily for any overt signs of infection. At day 7 p.i. the mice displayed no symptoms apart from a mild piloerection which continued throughout the infection. By day 21 p.i. the mice had a swollen abdomen indicating an enlarged spleen. By day 28 p.i. the mice had a more severe piloerection, exhibited longer periods of inactivity and reduced co-ordination when active. The average survival time of BALB/c mice following *T.b. brucei* infection was 37.9 ± 1.2 days (*n* = 21). No parasites were detected in the CNS at day 7 p.i. One out of three recipient mice that were injected with brain taken from donors that had been infected for 11 days relapsed. This was the earliest time that viable parasites were detected in the CNS. By 13 days p.i. all donor mice had cerebral parasites and were able to establish patent infections in naïve recipients. Recipient mice relapsed earlier when day 21 p.i. donor mice were used compared with day 14 p.i.: blood parasites being detected at day 4 and days 7–14 respectively, in the recipient mice (*p* = 0.008; Student’s unpaired *t*-test).

### Effect of parasite on barrier integrity/permeability

**Fig. 3:**
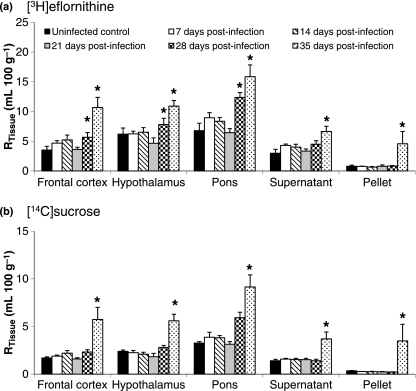
The effect of *T.b. brucei* infection on the *R*_Tissue_ values for (a) [^3^H]eflornithine and (b) [^14^C]sucrose in the brain regions and capillary depletion samples. Supernatant and pellet samples were obtained after capillary depletion analysis of brain homogenate. BALB/c mice were infected and perfused on days 7 through 35 p.i. Any statistical significance between the infected groups when compared with the non-infected group is noted. *p < 0.001. Each group *n* = 5–11.

When compared with uninfected mice, no differences were observed in the *R*_Tissue_ values for either [^3^H]eflornithine or [^14^C]sucrose measured in the brain regions of mice that were infected for 7 to 21 days with *T.b. brucei*. However, at day 28 p.i., the R_Tissue_ values for [^3^H]eflornithine in all brain regions were higher than those measured at day 7 p.i. (*p* = 0.007), day 14 p.i. (*p* = 0.025), day 21 p.i. (*p* < 0.001) and in uninfected mice (Fig. 3; *p* < 0.001). A further increase was also observed in the *R*_Tissue_ values achieved for [^3^H]eflornithine in the brain regions at day 35 p.i. compared with control mice (*p* < 0.001) as well as day 7 to day 28 p.i. (*p* < 0.001 for each time p.i.). Despite an apparent increase in the vascular/[^14^C]sucrose space measured in the pons at day 28 p.i., overall there was no statistical difference in [^14^C]sucrose accumulation in any brain region at this time-point compared with non-infected controls. In contrast, a significant increase was observed in all regions at day 35 p.i. when compared with uninfected mice (*p* < 0.001).

**Fig. 4:**
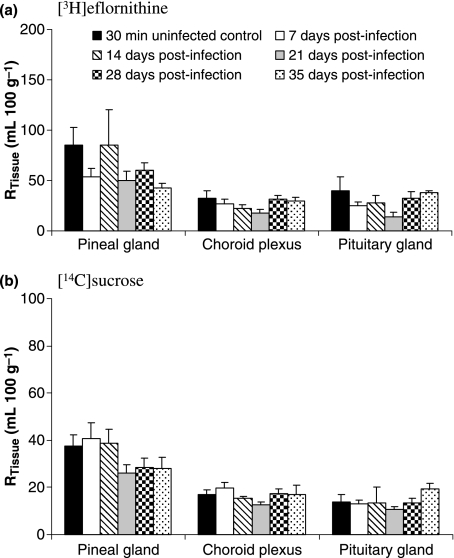
The effect of *T.b. brucei* infection on the *R*_Tissue_ values of (a) [^3^H]eflornithine and (b) [^14^C]sucrose in the CVOs. BALB/c mice were infected and perfused on days 7 through 35 p.i. No statistical difference was detected between infected and non-infected groups. Each group *n* = 5–11.

There was an increase in the *R*_Tissue_ levels of both [^3^H]eflornithine and [^14^C]sucrose in the capillary depletion samples at day 35 p.i., but not day 28 p.i., when compared to the control groups (*p* < 0.001 for both isotopes). This was most obvious in the pellet where respective *R*_Tissue_ levels were increased by 6 and 10 times those measured in uninfected mice (Fig. 3). In contrast, no significant differences were observed in the [^14^C]sucrose *R*_Tissue_ values in the CVOs at any time-point throughout the course of infection when compared to uninfected mice (Fig. 4). A small decrease was observed in the [^3^H]eflornithine detected in the CVOs at day 21 p.i. compared with uninfected controls (*p* = 0.029 across all CVOs). However, it is important to note that day 21 p.i. mice were not significantly different to other post-infection time-points. The choroid plexus weight of the non-infected group (0.322 ± 0.029 mg) was not significantly different from that measured in day 7 (0.270 ± 0.042 mg), 14 (0.337 ± 0.037 mg), 21 (0.289 ± 0.065 mg), 28 (0.362 ±0.058 mg) or 35 (0.432 ± 0.129 mg) p.i. groups.

### Octanol-saline partition coefficients and protein binding

The octanol-saline partition coefficient of [^3^H]eflornithine was 0.00487 ± 0.00010. No [^3^H]eflornithine was detected bound to bovine serum albumin or dextran in the artificial plasma or to protein in the mouse or human plasma.

## Discussion

Developed as an anti-cancer chemotherapeutic called difluoro-methyl-ornithine, eflornthine was subsequently shown to have anti-protozoal activity (Bacchi *et al.* 1980; McCann *et al.* 1981; Van Nieuwenhove *et al.* 1985) and is now used in the treatment of stage 2 infection with *T.b. gambiense*. The fact that it is effectively used against the CNS-stage has led to the assumption that it readily crosses the BBB. However, our study, which directly investigates movement across the blood–CNS interfaces *in situ,* but in the absence of systemic metabolic influences, shows that eflornithine does not cross the healthy murine BBB well. This is further highlighted by comparing brain parenchyma (supernatant) unidirectional transfer constants (*K*_in_; determined by single-time analysis). Eflornithine (0.52 ± 0.18 μL/min/g) and pentamidine (stage 1 drug; 0.68 ± 0.12 μL/min/g) having similar values and nifurtimox (stage 2 drug 56.7 ± 10.6 μL/min/g) being greater (unpublished observations). Furthermore, prolonged exposure of eflornithine to the blood–CNS interfaces does not affect barrier integrity as measured by [^14^C]sucrose. This limited brain distribution of [^3^H]eflornithine has been observed after intravenous administration in healthy rats; permeability coefficients being similar for eflornithine (3.9 × 10^−7^ cm/s) (Levin *et al.* 1983) and the *non-permeating* molecule, sucrose (1.2 × 10^−7^ cm/s) (Levin *et al.* 1976). This is unsurprising, if one considers its hydrophilicity (octanol-saline partition coefficient of 0.00487 ± 0.00010). In fact, the ability of eflornithine to cross the BBB can be predicted from its physicochemical characteristics (Levin *et al.* 1983). Furthermore, eflornithine has a short half-life (3.3 h) and ∼80% is eliminated via the kidneys in healthy humans (Burri and Brun 2003). All these factors reduce the concentration of eflornithine that could be achieved in the brain. In agreement with this current study, eflornithine does not bind significantly to plasma proteins (Burri and Brun 2003) or undergo significant metabolism in mice (Romijn *et al.* 1987). Our study also demonstrates that eflornithine can cross the blood–CSF barrier, although the choroid plexus levels are low (16 ± 6 mL/100 g) in comparison with suramin (163 ± 32 mL/100 g) (Sanderson *et al.* 2007). Eflornithine can diffuse into the CSF of healthy volunteers (Burri and Brun 2003) and patients with *T.b. gambiense* infections (Doua *et al.* 1987; Taelman *et al.* 1987; Na-Bangchang *et al.* 2004), albeit at low levels (Na-Bangchang *et al.* 2004) and eflornithine treatment can clear parasites from CSF (Milord *et al.* 1992). Furthermore, intraventricularly infused eflornithine only leaves the ventricles by bulk absorption from the CSF (Levin *et al.* 1984), indicating that eflornithine movement from the CSF to brain is limited. This is also apparent in our study as higher levels of eflornithine were detected in the CSF compared with the brain samples.

Based on the experiments described in this present study we can estimate that, after a 30 min perfusion, eflornithine reaches a concentration of at least 7 nM in the murine brain. The IC_50_ value against bloodstream forms of *T.b. gambiense* is considerably higher being 10.7 μg/mL (59.0 μM) for eflornithine (Likeufack *et al.* 2006). However, it is important to note that eflornithine is a cytostatic rather than a trypanolytic drug and requires an intact immune system to elicit a cure. This is obviously lacking in the *in vitro* system used to determine IC_50_. Thus the IC_50_ value is likely to be an overestimate and it is impossible to say if therapeutic brain concentrations of [^3^H]eflornithine are achieved. However, as eflornithine has to be administered intravenously every 6 h for 14 days (100 mg/kg body weight) for it to be effective, it is reasonable to consider that this intensive regimen is, at least partly, a consequence of limited blood–brain and blood–CSF barrier penetration. Eflornithine induces the differentiation of trypanosomes from the long slender blood-stream forms to short stumpy non-dividing forms (Giffin *et al.* 1986). This inhibition of proliferation prevents further changes in the parasite antigenic repertoire thus making the trypanosome accessible to the immune system. The effectiveness of an intensive eflornithine regimen at treating stage 2 HAT may be related to the fact that different forms of the parasite cannot cross the BBB (Grab *et al.* 2004), than ensuring effective brain concentrations of eflornithine are reached. However, eflornithine is only administered once the parasites are detected in the CNS suggesting that the drug has to reach the CNS to cure the established CNS infection. Furthermore, one of the major determinants of successful eflornithine treatment seems to be the CSF drug level reached during treatment, and it was shown that levels above 50 μM must be reached to attain a consistent clearance of parasites (Burri and Brun 2003). This indicates that eflornithine movement across the BBB is an important prerequisite for successful treatment of HAT.

It has been suggested that eflornithine is actively or passively effluxed from the CNS (Na-Bangchang *et al.* 2004). Eflornithine is a cationic amino acid analogue and facilitated and active transport of cationic amino acids has been described at the BBB (O’Kane *et al.* 2006). However, the physicochemical characteristics of eflornithine, together with our multiple-time and self-inhibition studies, suggest that a transporter is not involved in the movement of this molecule into or out of the murine brain. Eflornithine enters murine fibroblasts and bloodstream trypanosomes by passive diffusion (Bitonti *et al.* 1986; Delespaux and de Koning 2007). P-glycoprotein is an efflux transporter expressed at the BBB, and hypothesized to contribute to the synergistic effect of drugs when they are used in combination. Our studies using mice that do not express P-gp, failed to produce any changes in the distribution of [^3^H]eflornithine. Eflornithine appears to cross the blood–CNS interfaces by diffusion.

In order to simplify treatment, shorten its duration and to avoid the development of parasite resistance against eflornithine, application in combination with the other anti-trypanosomal drugs is being considered(Priotto *et al.* 2007). A combination of eflornithine and suramin proved effective in curing CNS animal models of *T.b. rhodesiense* (Bacchi *et al.* 1994) *and T.b. brucei* infections (Clarkson *et al.* 1984; Bacchi *et al.* 1987), although the drugs when used alone were ineffective (Bacchi *et al.* 1994). The mechanism resulting in this potentiation is unknown. It is therefore of interest that unlabelled suramin increased the measured [^3^H]eflornithine in all brain regions in particular the cerebral capillary endothelial cells in this study. Clearly this could explain the synergistic effect of eflornithine and suramin combinations. As previously stated the presence of unlabelled eflornithine had no effect on [^3^H]eflornithine distribution. Furthermore, our earlier study confirmed that eflornithine or suramin had no effect on [^3^H]suramin CNS distribution (Sanderson *et al.* 2007). However, evidence of suramin interacting with plasma membranes was found (Sanderson *et al.* 2007). Taken together these results indicate that the interaction of [^3^H]eflornithine with suramin may be due to endocytosis of suramin and consequently eflornithine. There was no change in the CNS distribution of [^3^H]eflornithine when in combination with pentamidine, melarsoprol or nifurtimox.

This study also examined the integrity of the blood–CNS interfaces during the course of trypanosome infection using a murine model of trypanosomiasis. BALB/c mice infected with the stabilate *T.b. brucei* GVR 35 survived for ∼38 days. CD1 mice infected with this stabilate survive for at least 30 days (Jennings and Gray 1983; Jennings *et al.* 2002). BALB/c mice infected with the AnTAT 1/1 clone died 28–36 days (Schultzberg *et al.* 1988) and 30 ± 3 days p.i. (Namangala *et al.* 2000). Blood parasitaemia fluctuated and exhibited a similar profile to BALB/c mice infected with AnTAT 1/1 (Namangala *et al.* 2000). In this present study viable parasites were not detected in the brain at day 7 p.i., but by day 14 all mice had cerebral parasites. Thus, although *T.b. rhodesiense* parasites injected directly into the murine brain do not survive (Schmidt and Bafort 1987), *T.b. brucei* parasites that cross the BBB *in vivo* remain viable. Parasites reached the CNS from day 11. Interestingly, the integrity of the blood–CNS interfaces, as measured by [^14^C]sucrose, was not affected by the presence of cerebral parasites at day 14 or 21 p.i.. Furthermore, greater parasite numbers were present in the brain at day 21 compared with day 14 p.i.. Increasing numbers of cerebral parasites has been observed in mice infected with the AnTAT 1/1 clone (Amin *et al.* 2008). In agreement, by day 21 p.i. with *T.b. brucei* GVR 35 there are considerable trypanosome numbers within the CNS of CD1 mice (Jennings and Gray 1983). This indicates that either parasites multiply within the CNS, fluctuating waves of parasites enter the CNS (possibly linked to the variable blood parasitemia) and/or parasites continually cross the blood–CNS interfaces during the course of infection. We found no evidence for an increase in BBB permeability at the early time-points, which implies that the parasites do not irreversibly damage the tight junctional components or endothelial cells themselves. Interestingly the tight junction proteins, occludin and zonula occludens 1, are unaffected by *T.b. brucei* infections in rats (Mulenga *et al.* 2001). Furthermore, *T.b. rhodesiense* but not *T.b. brucei*, caused a transient change in BBB permeability as measured by transendothelial electrical resistance in a human brain microvessel endothelial cell line during an overnight parasite incubation (Grab *et al.* 2004; Nikolskaia *et al.* 2008). This implies a reversible change in paracellular permeability and tight junction integrity with human trypanosome invasion of the CNS.

Murine infection with *T.b. brucei* GVR 35 results in no neuropathological findings at day 7 p.i., a mild meningitis at day 21 p.i. and a moderate meningitis at day 28 p.i. (Jennings and Gray 1983). In this study, trypanosome infection only started affecting the functional integrity of the BBB, as measured by [^14^C]sucrose, late in the infection course (day 28). This finding supports the clinical evidence that drugs that pass the BBB are required for the treatment of second stage HAT (Van Nieuwenhove 1999). Interestingly, the smaller molecule, eflornithine (MW237), was able to cross more rapidly than sucrose (MW342) at day 28. By day 35 p.i. there was an even greater loss of BBB integrity with significant increases in both [^14^C]sucrose and [^3^H]eflornithine R_Tissue_ in all brain regions. This confirms a report of substantial BBB breakdown in the late phase of *T.b. brucei* infection in rats (Philip *et al.* 1994) and also suggests a gradual loss of BBB integrity with time rather than a complete breakdown. Importantly, although the pons appeared more fragile to this breakdown, statistically no region was more sensitive to this loss of integrity at either day 28 or 35 p.i.. Thus we were unable to demonstrate a direct link to the classical HAT complication of white matter encephalitis (Kennedy 2004). The [^3^H]eflornithine and [^14^C]sucrose concentrations in the capillary endothelial cell-enriched pellet in the day 35 p.i. mice was increased compared to the non-infected and other infected time groups. This cannot be explained by an increase in the paracellular transport of the molecules due to a loss of tight junctional integrity. However, it may reflect an increase in vesicular trafficking within the endothelial cells.

There was no evidence of blood–CSF barrier dysfunction in terms of extracellular space changes (e.g. oedema) in the fourth ventricle choroid plexuses throughout the infection. Values being 13–20% in both uninfected and infected animals, which is similar to that measured in healthy rats (Smith *et al.* 1981). Oedema does occur in rabbit choroid plexus during trypanosome infection (Ormerod and Segal 1973). However, choroid plexus abnormalities were focal and were inconsistent between lateral and fourth ventricle in rodent *T.b. gambiense* and *brucei* infections (Van Marck *et al.* 1981; Quan *et al.* 1999). Furthermore, blood–CSF barrier impairment is not a permanent feature of *T.b. rhodesiense* infections in vervet monkeys (Waitumbi *et al.* 1988). Importantly, choroid plexus oedema has been linked to blood–CSF permeability disruption in animal models of ischemia (Ennis and Keep 2006). The increased fluid volume distending the choroidal epithelium making the structure more permeable (Murphy and Johanson 1985). Although it is unknown if trypanosomes induce ischemia, it is noted that whilst the choroid plexus epithelium is extremely sensitive to ischemia and undergoes significant morphological and functional damage, it does recover quickly (Johanson *et al.* 2000). This recovery may reflect the rearrangement of the surviving cells to create a smaller, but functional plexus or new epithelial cells being produced (Keep and Ennis 2005). However, the choroid plexus weight remained unchanged during the trypanosome infection suggesting that a damage-recovery process had not occurred or was undetectable at the time-points measured.

CSF samples are difficult to take in small animals and can be contaminated by blood or artificial plasma. Hence only crystal-clear samples from the healthy animals were taken for radioactive analysis. [^14^C]sucrose CSF levels were also used to highlight non-visible contamination and samples to be discarded. In the infected animal model, contamination and high [^14^C]sucrose levels may indicate blood–CSF barrier breakdown. Although it was noticeable that the CSF samples became more difficult to obtain as the infection progressed, the CSF results from the infected animals are impossible to interpret accurately and we cannot draw any conclusion in terms of changes to CSF drug penetration during the infection. In a clinical study the highest drug levels were obtained with the most severe disease form, suggesting a penetration into the CSF proportional to the degree of CNS involvement (Taelman *et al.* 1987). This may reflect BBB rather than blood–CSF barrier breakdown. In fact, CSF/serum ratios of eflornithine are higher in patients with melarsoprol-refractory infections, possibly because of severe BBB impairment leading to increased permeability as a consequence of chronic meningoencephalitis (Burri and Brun 2003).

To summarize this study highlights the misconception that eflornithine crosses the blood–brain and blood–CSF barriers well. In fact the intensive administration schedule required for this drug to be effective is probably partly due to its inability to rapidly cross the BBB. Eflornithine crosses the blood–CNS interfaces by diffusion and eflornithine entry into the CNS can be enhanced with suramin. This explains the observed synergy of eflornithine-suramin combinations in CNS efficacy models and is the first to demonstrate that combination therapy can prove efficacious due to enhanced drug delivery to the CNS. Furthermore, this study also illustrates that parasites reach the CNS early in the course of infection, irreversible blood–brain and blood–CSF barrier breakdown is unnecessary for parasites to reach the CNS, parasites that cross the BBB *in vivo* remain viable, and widespread BBB dysfunction occurs during the terminal stage of the disease.

## Abbreviations used

BBB: blood–brain barrier
CVOs: circumventricular organs
HAT: human African trypanosomiasis
p.i.: post-infection
PBS-G: phosphate-buffered saline-containing glucose
P-gp: P-glycoprotein
*T.b.*: *Trypanosoma brucei*
